# Effective Antimicrobial Solutions for Eradicating Multi-Resistant and β-Lactamase-Producing Nosocomial Gram-Negative Pathogens

**DOI:** 10.3390/antibiotics10111283

**Published:** 2021-10-21

**Authors:** Elaine Meade, Micheal Savage, Mary Garvey

**Affiliations:** 1 Department of Life Science, Sligo Institute of Technology, Ash Lane, Sligo, Ireland; elaine.meade@mail.itsligo.ie; 2 Lir Analytical Ltd., Century Business Park, Unit 2, Athlone Rd, Longford, Ireland; micheal.savage@liranalytical.com

**Keywords:** multi-drug resistant, nosocomial pathogens, 1,10-phenanthroline-5,6-dione, intermediate-level disinfectants

## Abstract

Antimicrobial resistance (AMR) remains one of the greatest public health-perturbing crises of the 21st century, where species have evolved a myriad of defence strategies to resist conventional therapy. The production of extended-spectrum β-lactamase (ESBL), AmpC and carbapenemases in Gram-negative bacteria (GNB) is one such mechanism that currently poses a significant threat to the continuity of first-line and last-line β-lactam agents, where multi-drug-resistant GNB currently warrant a pandemic on their own merit. The World Health Organisation (WHO) has long recognised the need for an improved and coordinated global effort to contain these pathogens, where two factors in particular, international travel and exposure to antimicrobials, play an important role in the emergence and dissemination of antibiotic-resistant genes. Studies described herein assess the resistance patterns of isolated nosocomial pathogens, where levels of resistance were detected using recognised in vitro methods. Additionally, studies conducted extensively investigated alternative biocide (namely peracetic acid, triameen and benzalkonium chloride) and therapeutic options (specifically 1,10-phenanthroline-5,6-dione), where the levels of induced endotoxin from *E. coli* were also studied for the latter. Antibiotic susceptibility testing revealed there was a significant association between multi-drug resistance and ESBL production, where the WHO critical-priority pathogens, namely *E. coli*, *K. pneumoniae*, *A. baumannii* and *P. aeruginosa*, exhibited among the greatest levels of multi-drug resistance. Novel compound 1,10-phenanthroline-5,6-dione (phendione) shows promising antimicrobial activity, with MICs determined for all bacterial species, where levels of induced endotoxin varied depending on the concentration used. Tested biocide agents show potential to act as intermediate-level disinfectants in hospital settings, where all tested clinical isolates were susceptible to treatment.

## 1. Introduction

Gram-negative bacteria (GNB) are a significant public health concern due to their nosocomial transmission and high levels of antibiotic resistance, where patients often require admittance to intensive care units (ICU), leading to high morbidity and mortality [1]. Clinically, two major groups, the Enterobacteriaceae and non-fermenters (opportunistic species) are responsible for the majority of HAIs; however, other clinically important GNBs including *Neisseria*, *Helicobacter pylori*, and *Psychrobacter* must also be considered [1]. Nosocomial or health care-associated infections (HAIs) are infections acquired by persons admitted to health care facilities, where they represent the greatest threat to patient safety [2]. The three main sites of HAIs are the urinary tract, bloodstream (septicaemia) and respiratory system (pneumonia), with ventilator-associated pneumonia frequently requiring admission to ICU [3]. The skin and other organs are also sites of infection though less frequently. HAIs associated with antimicrobial-resistant (AMR) and multi-drug-resistant (MDR) bacterial species result in increased morbidity, mortality, and socio-economic impacts on patients. Studies report a prevalence of 8% of hospitalised patients developing HAIs, with 20% of these involving MDR species having a higher risk of mortality or re-admission [4] The European Centre for Disease Prevention and Control (ECDC) estimates approximately 8.9 million HAIs yearly in Europe, with the Gram-negative *Pseudomonas aeruginosa* and the Enterobacterale *Escherichia coli* prominent agents of disease [5]. Importantly, Gram-negative non-fermenting species such as *P. aeruginosa* are intrinsically resistant to common antibiotics such as ampicillin, cephalosporins, and macrolides, where MDR is common [6]. The extended-spectrum β-lactamase enzymes (CTX-M, TEM, SHV, PER, VEB, and TLA) which hydrolyse extended-spectrum cephalosporins and penicillins are abundant in GNB and are plasmid transferrable, promoting pan-resistance in species [1]. Other Gram-negative pathogens associated with HAIs include the Enterobacterale species, *Proteus mirabilis*, *Klebsiella pneumoniae* and *Citrobacter freundii*, and non-Enterobacterale species, *Acinetobacter baumannii*, *A. lwoffii*, *Pseudomonas fulva* and *P. fluorescens*. High levels of AMR in sexually transmitted *Neisseria gonorrhoeae* is also an emerging global health threat, where the WHO lists antibiotic-resistant *N. gonorrhoeae* as a high-priority pathogen [7]. Pathogenesis and disease progression are dependent on an interplay between bacterial virulence factors and host factors such as immunity, age and nutritional status. Virulence factors promote bacterial colonisation and pathogenesis, where microbial species have evolved mechanisms to invade host cells, evade host immunity and reproduce intracellularly. GNBs possess an array of chromosomal located and acquired factors including adhesins, invasins, outer membrane proteins, toxins such as the lipopolysaccharide (LPS) toxin, capsules, AMR genes and biofilm capabilities [8] amongst others. The LPS endotoxin of Gram-negative species which is released following cell lysis or proliferation is of particular concern due to the development of sepsis or septic shock in patients. The presence of LPS toxin is correlated with cardiovascular failure, acute physiology and chronic health evaluation and organ failure in patients [9]. As antibiotic therapy options become increasingly limited for these GNB, prevention of transmission is essential to reduce the spread of such high-risk organisms. Alarmingly, the presence of biocide resistance has emerged in AMR species, where sublethal biocidal exposure proliferates resistance in numerous species [6]. For example, evidence of resistance to quaternary ammonium compounds (QACs) and benzalkonium chloride (BAC) in MDR species due to the presence biocidal resistance genes (BRGs) has emerged, including the *qacE* and *qacA/B* genes common in the Enterobacteriaceae family and *Pseudomonas* species [10]. The assessment of novel low-level and high-level biocidal options is essential to provide alternative or combination disinfectant options for use in health care settings. This study determines the resistance profile of numerous GNB of clinical importance and assesses their susceptibility to potential alternative antimicrobial compounds where LPS release is also considered.

## 2. Methodology

Reagents including all antibiotics and 1,10-phenanthroline-5,6-dione (phendione) were sourced from VWR, Dublin, Ireland. All therapeutic agents were made to stock solutions by dissolving the drug powder in adequate volumes of the recommended diluent, i.e., polar compounds were dissolved in sterile water while non-polar compounds were dissolved in dimethyl sulfoxide (DMSO) (*w*/*v*). Once completely dissolved with no precipitation evident, working concentrations of all test agents were then prepared based on EUCAST recommendations 2021. In addition, gradient concentration Liofilchem^®^ MIC test strips (MTS) (Launch Diagnostics, Sligo, Ireland) encompassing the various antibiotic agents were used.

### 2.1. Bacterial Collection, Identification, Culture, and Maintenance

Bacterial pathogens including *Acinetobacter baumannii*, *A. beijerinckii*, *A. lwoffii*, *Pseudomonas aeruginosa*, *P. fulva*, *P. fluorescens*, *Escherichia coli*, *Klebsiella pneumoniae*, *Citrobacter freundii*, *Proteus penneri*, *Proteus mirabilis*, and *Psychrobacter pulmonis* were donated by registered clinical personnel in sterile containers (Cruinn Diagnostics, Dublin, Ireland), where the Neisseria gonorrhoea isolate was donated from Cork University Hospital, Ireland. Collected samples were immediately inoculated onto nutrient agar Petri plates (Cruinn Diagnostics, Dublin, Ireland) and incubated at 37 °C for 24 h. The N. gonorrhoea isolate was cultured on chocolate agar (compromised of GC agar base (Cruinn Diagnostics, Dublin, Ireland) supplemented with 2% haemoglobin, polyenrichment growth and antibiotic LCAT selective (Carl Stuart Ltd., Dublin, Ireland)) and incubated at 35–37 °C in 5% CO_2_. Individual colonies were re-streaked for isolation and pure isolated colonies inoculated into nutrient broth for further biochemical characterisation. Colonies were identified based on their morphological characteristics, biochemical profile, and growth on selective agars, specifically, Lab M’s Harlequin E. coli/Coliform Medium (Cruinn Diagnostics, Dublin, Ireland), Klebsiella ChromoSelect agar (Sigma Aldrich, Dublin, Ireland), and CHROMagar™ Pseudomonas and Acinetobacter agars (CHROMagar, Paris, France). Identity was confirmed via colony polymerase chain reaction (PCR) as per Meade et al. 2020 using bacterial primers ITS-8F 5′-AGAGTTTGATCCTGGCTCAG-3′ and ITS_U1492R 5′- GGTTACCTTGTTACGACTT-3′. Following DNA extraction and amplification, clean-up and gene sequencing of PCR products was completed by GATC biotech (London, UK). Strains were stored long term in 20% glycerol at −80 °C and short term in nutrient broth at 5 °C, where identity was confirmed via Gram stain and selective agars prior to each experimental set up.

### 2.2. Antimicrobial Susceptibility Testing

Antimicrobial susceptibility testing of the isolates was performed by the standard disk diffusion method as recommended by the European Committee for Antibiotic Susceptibility Testing (EUCAST). Bacterial inocula were prepared by suspending freshly grown colonies in phosphate buffer saline and adjusting to a density of 0.5 McFarland or ca. 1 × 10^8^ cells/mL. Mueller–Hinton agar (or chocolate agar) plates were inoculated with the resulting suspensions by introducing a sterile swab into the culture and streaking across the surface. The procedure was repeated two more times rotating the plate approximately 60 degrees each time to ensure an even distribution of inoculum. Antibiotic impregnated paper disks (Fisher scientific, Dublin, Ireland) were placed on the plate and incubated inverted for 24 h at 37 °C. Chocolate agar plates were enclosed in anaerobic jars and incubated at 35 °C in a 5% CO_2_-enriched environment. Zones of inhibition were measured in millimetres and antibiotic susceptibility results interpreted as Resistant I, Susceptible (S), or Susceptible, increased exposure (I) according to the EUCAST guidelines.

### 2.3. Phenotypic Detection of ESBL- and AmpC-Producing Isolates

Bacterial strains that showed resistance to at least one of extended cephalosporins tested were initially screened for ESBL production by inoculation on CHROMagar ESBL selective (CHROMagar, Paris, France). Isolates that displayed a positive result on the chromogenic media were selected for further confirmation of ESBL production using the combination disk method. Briefly, a bacterial lawn of the selected test strain was overlaid on freshly prepared Mueller–Hinton agar (or chocolate) agar plates using a sterile cotton swab. Pairs of antibiotic disks containing cefpodoxime (10 µg/disk) alone and cefpodoxime in combination with clavulanate (10:1 µg/disk) were placed on opposite sides of the same inoculated plate, approximately 30 mm apart. Plates were inverted and incubated overnight under appropriate conditions. Test organisms were considered positive for ESBL production if the zone diameter around the combination disk was at least 5 mm greater than that of the cephalosporin disk alone. Combination test strips containing ceftazidime (0.5–32 μg/mL) gradient at one end and ceftazidime (0.064–4 μg/mL) plus 4 μg/mL clavulanate gradient at the other were used to confirm ESBL production. In addition, strips containing cefotetan (0.5–32 μg/mL) and cefotetan (0.5–32 μg/mL) plus cloxacillin were used to detect AmpC production. The test was performed according to the manufacturer’s instructions. MICs were read at both ends of the strip at the point of intersection between the inhibition ellipse and the MIC scale of the strip. ESBL and AmpC production was inferred if the MIC ratios for the antibiotic alone compared with the antibiotic plus inhibitor was ≥8.

### 2.4. Genotypic Detection of ESBL- and AmpC-Producing Isolates

All isolates found to be phenotypically positive for β-lactamases were further assessed for the presence of *bla_TEM,_ bla_SHV_, bla_CTX-M1,_ bla_CTX-M2_* and *bla_AmpC_* genes by PCR using specific primers (see Table S1 in the Supplementary Materials). Bacterial DNA of selected isolates was extracted using the boiling method. PCR was performed in a total reaction volume of 20 µL, containing 17 µL red Taq 1.1x master mix (VWR, Dublin, Ireland) 1 µL of each primer and 1 µL of DNA (10 ng/µL). DNA amplification was performed in a thermo cycler (VWR, Dublin, Ireland) using the recommended parameters. Clean-up and bidirectional sanger sequencing of PCR products was completed by GATC (London, UK).

### 2.5. Minimum Inhibitory Concentration (MIC)

MICs were determined using the microdilution method, agar dilution method and gradient test strips. MIC results were subsequently compared to standardised EUCAST clinical breakpoints, where species were classed as Resistant (R) Susceptible (S) or Susceptible, increased exposure (I).

#### 2.5.1. Microdilution Method

The microdilution assay was performed in 96-well plates using Mueller–Hinton broth (Cruinn Diagnostics, Dublin, Ireland). Testing was conducted according to the EUCAST guidelines, where final well concentrations ranged from 0.01 to 256 µg/mL for antibiotic agents and 0.19 to 100 µg/mL for phendione. Briefly, antimicrobial test solutions were prepared at 2× the final concentration in Mueller–Hinton media and 100 µL added to the respective well of the 96-well microtiter plates, being arranged in twofold dilutions from left to right and in triplicate. Control wells contained 100 µL sterile drug-free medium. Bacterial suspensions were prepared at a density of 0.5 McFarland and diluted 1:100 in Mueller–Hinton broth. Wells were then inoculated with 10 µL of the respective test suspension, including growth control wells, resulting in a final inoculum of approximately 5 × 10^5^ cfu/mL. Plates were incubated without agitation for 24 h at 37 °C. MICs were measured visually, being recorded as the lowest concentration at which bacterial growth was completely inhibited.

#### 2.5.2. Agar Dilution Method

Chocolate agar containing 0.19 to 100 µg/mL phendione was prepared in 90 mm plates in triplicate. A 0.5 McFarland suspension of *N. gonorrhoea* was prepared and diluted 1:10 to give 10^7^ cfu/mL. 1 µL of the suspension was inoculated on the prepared plates resulting in a final bacterial inoculum of 1 × 10^4^ cfu/spot. Plates were incubated overnight at 35 °C in 5% CO2. The MIC was read visually as the lowest concentration that completely inhibited visible growth, where a single colony or a thin haze within the area of the inoculated spot was disregarded.

#### 2.5.3. Gradient Strip Method

A 0.5 McFarland suspension was spread on chocolate agar plates and respective antibiotic MIC gradient strips applied (Launch Diagnostics, Sligo, Ireland). Plates were inverted and incubated overnight at 35 °C in 5% CO2. MICs were read visually where the inhibition ellipse intersects the MIC scale of the strip.

### 2.6. Endotoxin Release Studies

Sterile pyrogen-free plastic-ware was used for conducting endotoxin studies. *E. coli* cultures were prepared by inoculating two to three colonies from Mueller–Hinton agar plates into 20 mL amounts of warm (37 °C) Mueller–Hinton broth. The broths were incubated for 2 h at 37 °C to allow the test strain to enter the exponential growth-phase. The antibiotic ceftazidime and test agent phendione at 2×, 10× and 100× the MIC were then added, respectively, to the cultures, containing ca. 10^7^ cfu/mL. Viable counts and the amount of endotoxin in the culture media were measured at 0, 1.5 and 3 h. A culture without antibiotics acted as a growth control. To determine released endotoxin, 1 mL portions of the cultures or controls, drawn concomitantly to those for viable counts, were filtered through a pyrogen-free 0.2 pm pore (Fisher Scientific, Dublin, Ireland). The filtrates were serially diluted 10^3^–10^5^ fold in pyrogen-free water and frozen at −20 °C pending analysis for the endotoxin assay. 

#### Determination of Endotoxin

Analyses of endotoxin were performed in duplicate with the chromogenic limulus amoebocyte lysate (LAL) assay (Thermo Fisher Scientific, Dublin, Ireland). Specifically, 50 µL of the test samples were added to 50 µL of LAL solution in a prewarmed sterile microtiter plate. After a 15 min incubation period at 37 °C, 100 µL of chromogenic substrate (supplied with kit) was added to the samples and the plate incubated for a further 5 min at 37 °C. The reaction was then stopped with 50 µL of a 25% acetic acid solution (Sigma Aldrich, Dublin, Ireland). Finally, optical densities were measured at 405 nm. Appropriate standards of endotoxin (ranging from 0.1 to 1 EU/mL) and pyrogen-free water were also included on each plate. Endotoxin concentrations were determined by extrapolating the absorbance of the unknown sample against the generated standard curve.

### 2.7. Biocidal Testing

The antibacterial agents investigated in this study are pure biocides used as disinfectants alone or in commercial brands. The concentrations described are the concentration of the active component and include the concentration used following manufactures instructions.

#### 2.7.1. Kirby–Bauer Assay

The Kirby–Bauer assay was carried out to determine the effect of disinfectants on microbial species with the presence and absence of an interfering substance. Bacterial test organisms including *E. coli*, *K. pneumoniae*, *P. mirabilis*, *A. baumannii*, *P. aeruginosa*, *P. fulva* and *P. fluorescens* were grown on Mueller–HintonMueller–Hinton (Cruinn Diagnostics, Dublin, Ireland) agar in the presence of various antimicrobial impregnated filter paper disks. The presence or absence of growth around the disks is an indirect measure of the ability of that compound to inhibit the test organism. Agars were prepared as per manufacturer’s instructions in deionised water at room temperature and poured to a depth of 4 mm. Immediately before inoculation, media was checked to ensure it was moist but free of water droplets on the agar surface and the Petri dish lids. The test inocula were prepared from a pure culture grown in nutrient broth for 6 h. To determine the effect of an interfering substance, 1 mL of ca. 1 × 10^7^ microbial cells was added to 9 mL Bovine Serum Albumin (BSA) to give a working microbial count of 10^6^ cells in 3 g/L and 10 g/L BSA solution. Subsequently, 100 μL of 10^6^ cells/mL microbial suspensions were transferred onto replicate agar plates and spread with a sterile L-shaped spreader (Cruinn Diagnostics, Dublin, Ireland) to ensure even distribution across the agar surface. Filter disks (6 mm) were immersed in the test solutions at respective concentrations of 0.01%, 0.1% and 1% (*v*/*v*) peracetic acid and triameen for 15 s where excess solution was allowed to drip off the disk. Subsequently, the disk was placed in the centre of the inoculated plate. Plates were then inverted and incubated accordingly for each test species for 24 h. Zones of inhibition were then measured using a Vernier calliper in millimetres for each test chemical and each test organism.

#### 2.7.2. BS EN 1276

Suspension tests were conducted in accordance with the guidelines of the BS EN 1276 for antibacterial testing of chemical disinfectants that form a homogeneous physically stable preparation in hard water and are used in food, industrial, domestic, and institutional areas. For compliance with test method, test chemicals must achieve a 10^5^ bacterial cell reduction in treatment times less than 30 min. Bacterial test suspensions were prepared by seeding sterile nutrient broth with an isolated colony and incubating under rotary conditions (125 rpm) for 12 h at 37 °C. Cell counts were adjusted to 10^9^ cfu/mL with sterile PBS. Chemical test solutions were prepared as per manufacture instructions for use on site and at a concentration above and below this working concentration giving a respective range of 0.01%, 0.1% and 1% (*v*/*v*) peracetic acid and triameen. Prior to testing all reagents were equilibrated to the test temperature of 20 °C using a water bath. Subsequently 8 mL of the test product was transferred to a sterile container with 1 mL of sterile water. Afterward, 1 mL of microbial suspension containing 1 × 10^9^ bacterial cells was added. Additionally, 1 mL of interfering substance at 3.0 g/L BSA and 10 g/L BSA was added with subsequent incubation for 0–15 min with mixing in a 20 °C water bath. At set intervals of 5, 10 and 15 min, 1 mL of the test mixture was transferred into a tube containing 8 mL neutraliser (30 g/L polysorbate 80 + 3 g/L lecithine/l-a-phosphatidylcholine from egg yolk) (Sigma Aldrich, Ireland) and 1 mL of sterile water. Samples were mixed and incubated in the water bath for 5 min. After neutralisation 100 µL of this bacterial suspension was transferred onto agar plates in triplicate and incubated at 37 °C for 24 h. Surviving cells of treated organisms was counted to determine the level of bacterial inactivation following exposure to the test solutions compared to the untreated control (PBS).

### 2.8. Statistics

All the experiments were performed three times with three plate replicates for each experimental data point providing a mean result for each experimental batch. Average zone diameter was calculated for the Kirby–Bauer assay with standard deviation and significance levels at 95% confidence determined for each strain. For suspension testing the log_10_ reduction was calculated as the log reduction in viable cell numbers (cfu/mL) of the non-treated (N0) and treated (N) samples [log_10_ (N0/N)]. Student *t* tests were conducted to determine significance levels (*p* < 0.05) of bacterial susceptibility to treatment and levels of susceptibility or resistance between species investigated using Minitab 16 (Minitab Ltd., Coventry, UK).

## 3. Results

Table 1 shows the resistance profile of isolated clinical pathogens as determined by standard disk diffusion and PCR methods. A multi-drug resistance phenotype, based on resistance to at least three different antimicrobial classes, was observed in all test species. Of the 18 different antibiotics tested, the carbapenem and quinolone classes proved the most effective, whereas the penicillins, cephalosporins and monobactams were among the least active agents. The high rate of resistance towards these agents was attributable to the presence of β-lactamase genes, where 12 of the 13 isolates were confirmed as β-lactamase producers. Molecular genotyping revealed 10 of these isolates (including *A. baumannii, A. beijerinckii*, *A. lwoffii*, *P. aeruginosa*, *P. fulva*, *E. coli*, *K. pneumoniae*, *P. pulmonis*, *P. mirabilis* and *P. Penneri)* harboured the *bla_TEM_* gene, where 1 of these isolates (*E. coli*) additionally carried the *bla_AmpC_* gene and 1 isolate (*C. freundii*) carried the *bla_CTX-M1_* gene. None of the isolates were found to possess *bla_SHV_* or *bla_CTX-M2_* genes. *P. mirabilis*, *and P. penneri* were both specified as broad-spectrum β-lactamase producers, where species fell well within the susceptible category to extended-spectrum cephalosporins (≥24 mm), however, were resistant to penicillin agents and narrow spectrum cephalosporins (test agent not shown). Resistance to the aminoglycosides, macrolides and tetracyclines was also evident for these species, where they additionally showed reduced susceptibility to chloramphenicol and carbapenem agents, where *Proteus* spp. possess intrinsic decreased susceptibility to imipenem. *E. coli*, *K. pneumoniae*, *C. freundii*, *P. pulmonis*, *Pseudomonas* spp. and *Acinetobacter* spp. were all identified as ESBL producers, where resistance to penicillins, one or more third-generation cephalosporin and aztreonam was evident. ESBL producers further displayed the highest levels of resistance to non-β-lactam agents, where *P. aeruginosa* was the most resistant species, exhibiting pan drug resistance PDR (resistance to all antibiotic agents). Extensive drug resistance XDR (susceptibility to 2 or fewer antimicrobial classes) was present in three isolates, where *K. pneumoniae* was only susceptible to the carbapenems (≥25 mm), and *C. freundii* and *P. fulva* showed decreased susceptibility (categorised as ‘I’) to the carbapenems and quinolones. *P. fluorescens* showed reduced susceptibility to the carbapenems (≤21 mm), quinolones (≤25 mm) and doxycycline (19 mm), where all other test agents produced inadequate zones (≤16 mm). Notably, *P. fluorescens* was the only ESBL-positive species to not harbour any of the β-lactamase genes screened for in this study (*bla_TEM_*, *bla_SHV_*, *bla_CTX-M1_*, *bla_CTX-M2_*, *bla_AmpC_*), where phenotypic data suggest other ESBL variants exist, where the isolate was screen positive in all ESBL confirmatory tests. The simultaneously presence of AmpC in the ESBL-producing *E. coli* strain resulted in resistance to the ESBL inhibitor, where no zone was observed for amox/clav. *E. coli* also demonstrated resistance to the aminoglycosides, macrolides, tetracyclines and polymyxins, where the most effective agents were the carbapenems (≥20 mm), quinolones (≥24 mm) and to a lesser extent chloramphenicol (18 mm; clinical breakpoint = 17 mm). Of the 3 *Acinetobacter* species, the WHO critical *A. baumanii* isolate proved the most resilient, showing resistance to streptomycin (13 mm), chloramphenicol (9 mm), colistin (13 mm) and doxycycline (10 mm). The isolate was, however, susceptible to the carbapenems (≥25 mm) and quinolones (≥30 mm), where a zone of 16 mm was evident for azithromycin. *A. beijerinckii* and *A. lwoffii* were more sensitive isolates, being susceptible to the aminoglycosides (≥19 mm), chloramphenicol (≥21 mm), macrolides (≥16 mm), carbapenems (≥20 mm) and quinolones (≥16 mm), where resistance was evident for colistin and doxycycline (≤14 mm). The ESBL-producing *P. pulmonis* isolate demonstrated resistance to the aminoglycoside, chloramphenicol, and macrolide agents, where zone diameters were ≤14 mm. While standardised breakpoints are lacking for *Psychrobacter* spp., the large zone sizes indicated the isolate was susceptible to the carbapenems (≥25 mm). However, reduced sensitive to quinolones (19 mm) was evident for this species when extrapolating breakpoints (22 mm) for other GNB. *N. gonorrhoea* was the only isolate not to harbour β-lactamase genes and showed the lowest levels of resistance to tested antibiotics, being susceptible to all β-lactam agents (≥27 mm), the quinolones (≥26 mm), and chloramphenicol (29 mm). However, resistance towards the macrolides and aminoglycosides was observed, where species harbour intrinsic resistance to polymyxin agents.

The MICs obtained for the various antibiotics and test agent phendione are presented in Table 2, where MICs are accompanied by EUCAST interpretive categories (i.e., Susceptible, Susceptible-dose dependent, or Resistant) when applicable. Results aligned with the disk diffusion assay, with the highest incidence of β-lactam resistance observed for amoxicillin (92%, 12/13), followed by the cephalosporins (76%, 10/13). *N. gonorrhoea* was the only isolate to remain sensitive to amoxicillin, with an MIC of 0.016 µg/mL, where no β-lactamase genes were detected. The MICs for the extended-spectrum cephalosporins varied, where non-ESBL-producing strains (*P. penneri* and *P. mirabilis* and *N. gonorrhoea*) were highly susceptible, with MICs ≤ 0.01, whereas all ESBL-producing species yielded typical cephalosporin-resistant MICs, with cefepime (fourth-generation agent) proving more active than ceftazidime and ceftriaxone (third generation), providing MICs for *A. lwoffii* (1 µg/mL), *K. pneumoniae* (0.75 µg/mL), and *E. coli* (0.5 µg/mL). Similar patterns were evident for the monobactam agent, where all ESBL-producing strains were resistant to aztreonam, while non-ESBL producers remained highly sensitive (MICS ≤ 0.047). The β-lactamase inhibitor-combination (amox/clav) was active against *P. penneri, P. mirabilis*, *N. gonorrhoea, P. pulmonis,* and *A. beijerinckii*, with MICs ≤ 2 µg/mL. *E coli* was deemed resistant to amox/clav (owing to the presence of AmpC gene), while *Pseudomonas* species and *K. pneumoniae* possess intrinsic resistance to amoxicillin. A high percentage of isolates were further resistant to the macrolides (76%, 10/13), chloramphenicol (76%, 10/13), doxycycline (54%, 7/13), and colistin (92%, 12/13), where proposed breakpoints of ≥32 µg/mL for azithromycin and ≥16 µg/mL for doxycycline were implied as no current clinical breakpoints exist. The *Acinetobacter* species were the only susceptible isolates to the macrolide agents, with azithromycin being more active than erythromycin, providing MICs that ranged from 1 to 4 µg/mL. Chloramphenicol was active against *N. gonorrhoea* (4 µg/mL), *A. lwoffii* (6 µg/mL), and to a lesser degree *E. coli,* where a MIC of 8 µg/mL is the cutoff for susceptibility. Majority of isolates exhibited elevated MICs for doxycycline and colistin, where *A. lwoffii* was the most sensitive species, being the only isolate susceptible to colistin, with MICs ≤ 2 µg/mL. Six of the isolates had MICs <16 µg/mL for doxycycline, where *A. lwoffii* (0.5 µg/mL) and *N. gonorrhoea* (4 µg/mL) were notably more sensitive than *P. fluorescens* (6 µg/mL), *A. beijerinckii* (6 µg/mL), *E. coli* (12 µg/mL), and *A. baumanii* (12 µg/mL), with MICs ≥ 4 µg/mL deemed susceptible-dose dependent (I). Resistance to doxycycline was observed for *P. aeruginosa*, *P. fulva*, *C. freundii* and *P. pulmonis* where MICs ranged from 24 to 64 µg/mL, while *Proteus* spp. possess intrinsic resistance to both doxycycline and colistin. The carbapenems and quinolones were the most active agents against the clinical isolates, with susceptibility rates of 85% (11/13) and 77% (10/13), respectively. Two isolates, *P. aeruginosa* and *C. freundii* showed resistance to the carbapenem agents, where MICs were elevated at least 2-fold above standard clinical breakpoints for doripenem and meropenem. Both *P. aeruginosa* and *K. pneumoniae* showed complete resistance to ciprofloxacin, where *P. pulmonis* exhibited decreased susceptibility, with a MIC of 2 µg/mL elevated above standard breakpoints of 0.5 µg/mL for other GNB. On the other hand, all test species were susceptible to phendione, where MICs ranged from 0.39 to 15 µg/mL, with the order of susceptibility (from most susceptible to least susceptible) as follows; *A. lwoffii*, *P. pulmonis*, *C. freundii*, *N. gonorrhoea* (0.39 µg/mL), *A. baumanii* (0.78 µg/mL), A. *beijerinckii* (1.56 µg/mL), *P. fluorescens* (3.125 µg/mL), *P. penneri*, *P. mirabilis*, *E. coli*, *K. pneumoniae* (6.25 µg/mL), *P. fulva* (6.25 µg/mL), and *P. aeruginosa* (15 µg/mL).

**Figure 1:**
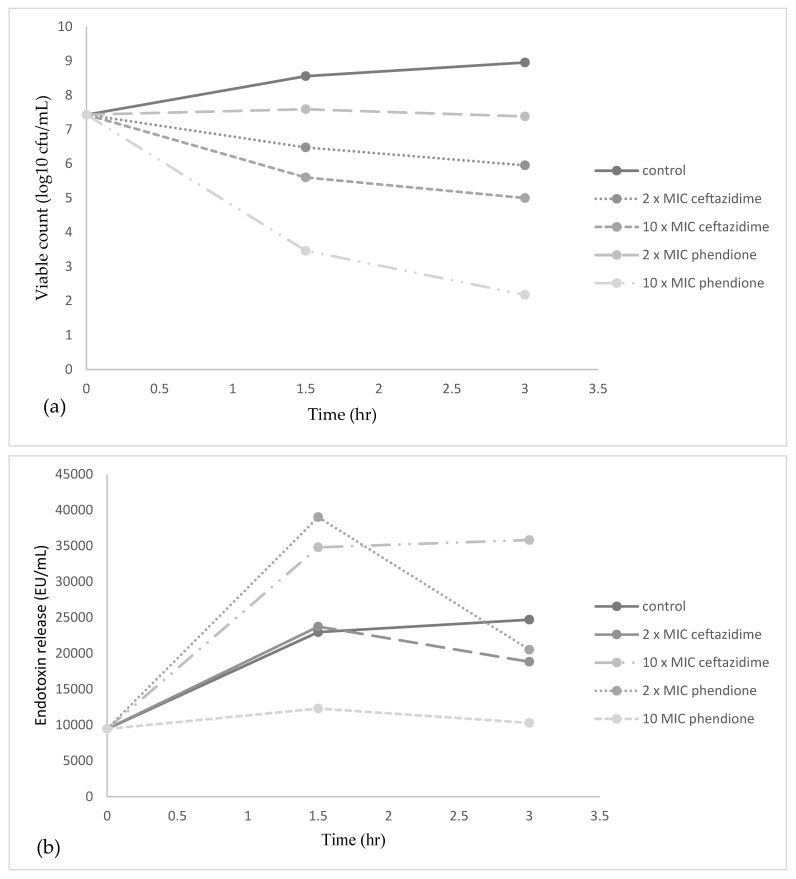
Effects of ceftazidime and phendione at 2× and 10× MIC on (**a**) growth and (**b**) release of endotoxin from ESBL *E. coli*, where drugs were added at time 0. Control cultures contained sterile media only.

The viable counts and endotoxin release data for *E. coli* following treatment with ceftazidime and phendione at 2× MIC, 10× MIC and 100× MIC are presented in Table 3 and Figure 1. To note, exposure of *E. coli* to ceftazidime or phendione at MIC concentrations gave no significant reduction in cell growth compared with that of the control, and thus this concentration level was excluded from further analysis. The results show that both ceftazidime and phendione (at levels elevated above the MIC) caused a rapid decline in viable cell counts within 3 h, where higher drug doses achieved greater levels of cell death.

In control cultures, endotoxin was released gradually from *E. coli* during logarithmic growth (<10,000 EU/mL) and was released in greater amounts during stationary phase (24,700 EU/mL). There was a marked increase in endotoxin release from *E. coli* compared to the control (22,962 EU/mL) following 1.5 h exposure to 2× MIC ceftazidime and phendione, reaching levels of 23,729 and 39,012 EU/mL, respectively. These levels of endotoxin decreased significantly to 20,506 EU/mL for phendione at 3 h, where a slight decrease to 18,824 EU/mL was recorded for ceftazidime at 2× MIC. When concentrations were increased to 10× MIC, endotoxin release was significantly greater for ceftazidime when compared with phendione, where recorded EU/mL after 3 h were 35,820 and 10,300 EU/mL, respectively. Noticeably in some instances, the amount of endotoxin released appeared to be significantly higher for control cultures when compared to treatment-exposed bacteria. However, when bacterial numbers were taken into account, the amount of liberated endotoxin from *E. coli* cells still remained lower for control cultures. The rates of endotoxin released per bacterium at 2× MIC phendione remained relatively low, being similar to those observed for control cultures. However, at 2× MIC ceftazidime, greater levels of endotoxin per bacterium were evident. At higher concentrations of ceftazidime (10× MIC) and phendione (10× MIC) the levels of endotoxin released per bacterium significantly increased (>36 EU/cfu) compared to control cultures (2.7 × 10^−5^ EU/cfu), where a concentration of 100 µg/mL (100× MIC) ceftazidime induced even greater levels (up to 384 EU/cfu).

Table 4 depicts the activity of disinfectant agents, peracetic acid, triameen and BAC, where various formulations are routinely used in the health care sector. All test strains proved susceptible to all three agents at concentrations recommended by the manufacturer. Peracetic acid was the most effective test agent, providing significant zones of inhibition that ranged from 18 to 26 mm at a concentration of 0.1% and 38 to 45 mm at 1%. The order of susceptibility among clinical isolates (from highest to lowest) was as follows: *P. mirabilis* (45 mm), *P. aeruginosa* (44.3 mm), *A. baumannii* (41 mm), *K. pneumoniae* (40 mm), *E. coli* (39 mm), *P. fulva*, and *P. fluorescens* (38 mm). The next most active agent was triameen, being most effective against *P. mirabilis* and *P. fluorescens* at all tested concentrations, where max zones of 33 and 30 mm were achieved, respectively. For all remaining test species triameen provided zones of inhibition ≥14 mm at 0.1% and ≥20 mm at 1%. BAC while effective, presented as the least active agent, requiring concentrations of 1% to produce zones analogous to those generated at 0.1% with peracetic acid and triameen. The non-fermenters appeared to be the most sensitive isolates to this agent, where zones of 20.5, 19.3, 16 and 14.5 mm are evident for *A. baumannii*, *P. fluorescens*, *P. fulva* and *P. aeruginosa* at a concentration of 1%. *K. pneumoniae* was the least sensitive isolate, where a max zone of 9.8 mm was provided at this same concentration. At a concentration of 0.l% BAC, the order of susceptibility of test strains from most sensitive to least was as follows: *A. baumanii* (14 mm), *P. fluorescens* (11 mm), *P. fulva* (10 mm), *P. aeruginosa* (10 mm), *E. coli* (9 mm), *P. mirabilis* (8.5 mm) and *K. pneumoniae* (7 mm). When the test was repeated with BSA, the bactericidal activities of disinfectants remained relatively constant for *E. coli*, *K. pneumoniae*, *A. baumannii*, and *P. fulva.* The presence of BSA did, however, influence the zone diameters for *P. mirabilis* and *P. fluorescens*, where the activity of peracetic acid and triameen were reduced significantly at concentrations of 0.1 and 1%. *P. aeruginosa* was the most affected isolate in the presence of BSA, where zone diameters were significantly reduced for all test agents.

**Figure 2:**
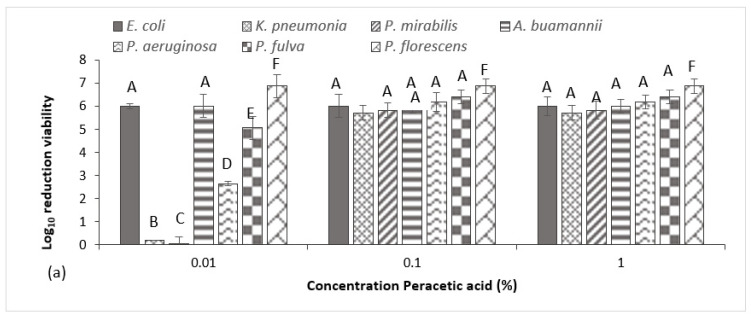

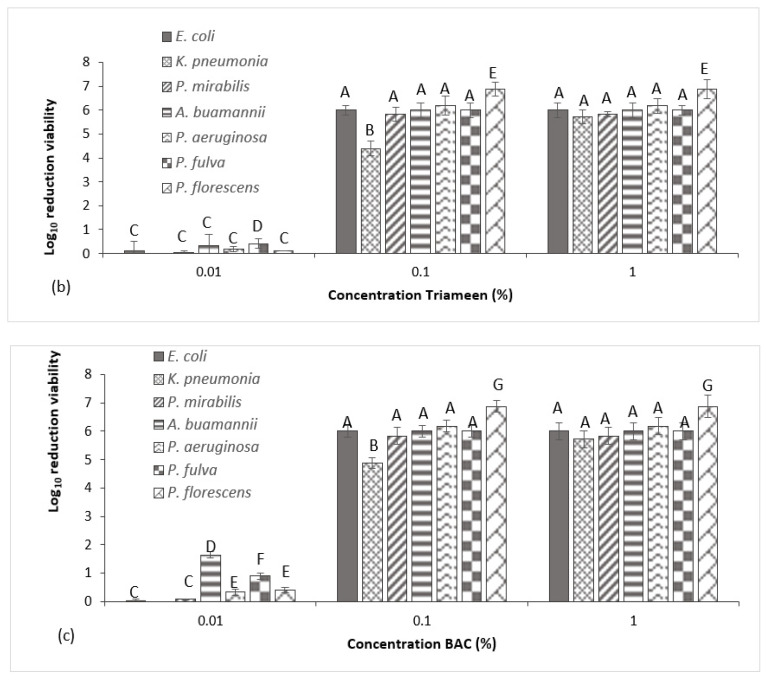
Log_10_ reduction in viable cfu/ml of test species as determined by BSEN 1276 following 5 min exposure to 0.01%, 0.1% and 1% (**a**) peracetic acid (**b**) triameen and (**c**) BAC in high interfering solution 10 g/L BSA and yeast extract (+/− Standard deviation). A, B, C, D, E, F and G denote a significant difference between the strains, where groups displaying the same letter are statistically similar whilst those denoted by a different letter are statistically different.

Results of the BS EN 1276 show all three disinfectants demonstrated rapid bactericidal activity under clean (data not shown) and enhanced dirty conditions (Figure 2), generating a >5log reduction for all strains within 5 min and therefore meeting relevant European testing requirements. Peracetic acid was the most effective test agent, where a concentration of just 0.01% provided the necessary 5 log_10_ inactivation for *E. coli*, *A. baumannii*, *P. fulva* and *P. fluorescens* (Figure 2a). For all remaining test species, a concentration of 0.1% peracetic acid was necessary to meet the test requirements, where no significant increase in inactivation was achieved when increasing the agent’s concentration to 1%. This was also true for triameen (Figure 2b) and BAC (Figure 2c), where a concentration equal and higher than 0.1% provided total bacterial inactivation within 5 min for all test species, except *K. pneumoniae*, which required an increased exposure time of 10 min for BAC to achieve equivalent results (data not shown). At a concentration of 0.01%, both triameen and BAC proved ineffective against all MDR isolates, failing to meet standard test requirements. In the presence of low interfering substance (data not shown) all test species proved to be more sensitive to test agents, indicating that increasing levels of interfering substance did impact on the test agent’s ability to inhibit test species. For triameen and BAC, the recommend user concentration of 0.1% was still required to meet test standards under clean conditions, whereas peracetic acid achieved complete inactivation of all test species at all tested concentrations within 5 min.

## 4. Discussion

The incidence of nosocomial disease caused by MDR Gram-negative pathogens continues to rise globally, where an exhausted antimicrobial pipeline now limits clinicians’ ability to provide safe and effective treatments for patients.

Of particular concern are infections caused by ESBL-producing bacteria, where treatment failure is high as species exhibit intense resistance to first-line β-lactam agents’, including extended-spectrum cephalosporins and monobactams. In the present study, the rate of MDR among isolated clinical pathogens was high, where all test species displayed resistance to at least one agent in three or more antimicrobial classes. Furthermore, this study reported a high prevalence of β-lactamase-producing bacteria (92%; 12/13 of isolates), with 10 isolates being identified as ESBL producers (Table 1). Among the ESBL- producers, resistance to ceftriaxone was observed in all 10 isolates, and resistance to ceftazidime and cefepime in 7 isolates, respectively. Multiple factors can contribute to apparent susceptibility in some species, including varying affinity of β-lactamase enzymes for different substrates, production of multiple β-lactamase genes by a single species, concomitant expression of AmpC and ESBL genes and inoculum effect [11].

This study found that the most prevalent genotype for β-lactamase production was *bla_TEM_*, which was detected in 10 strains, followed by *bla_CTX-M1_*, which was detected in *C. freundii*. One isolate, *E. coli*, was found to concurrently harbour *bla_AmpC_* and *bla_TEM_* genes. These results are consistent with global reports [12] where TEM (and SHV) variants are dominant among hospital isolates. Notably, the appearance of *bla_CTX-M1_* supports recent European studies that show there is a gradual shift from TEM and SHV mutants as the predominant ESBLs in Enterobacteriaceae to CTX-M-type [13]. The presence of AmpC, a less prevalent but increasingly relevant class of β-lactamases, presents an additional challenge, where unlike ESBLs, they mediate resistance to β-lactamase inhibitor- β-lactam combinations such as clavulanic acid, tazobactam and sulbactam, as seen in the present study. Factors that contribute to the increasing prevalence of ESBLs are complex and primarily involve the spread of mobile genetic elements (mainly conjugative plasmids), which in turn facilitates the spread of resistant bacterial clones between hospitals and communities via patient mobility [14]. In addition, the selective pressure imposed by the indiscriminate use of third-generation cephalosporins continues to promote the emergence of new variants of ESBLs, where inhibitor-resistant TEM, SHV and CTX-M-type enzymes have now evolved.

Currently, the medical literature abounds with studies demonstrating the global increase in multi-drug-resistant ESBL-producing strains, where they have risen to prominence among Enterobacteriaceae isolates in both hospital and community infections [12]. In the present study, ESBL-producing isolates displayed co-resistance to multiple non-β-lactam agents, including macrolides, chloramphenicol, doxycycline and colistin (Table 2). Fluroquinolone (FQ) susceptibility varied among ESBL producers, where two isolates (*E. coli* and *A. lwoffii*) were fully susceptible to ciprofloxacin, five isolates (*A. baumannii*, *A. beijerinckii*, *P. fulva*, *P. fluorescens* and *C. freundii*) were intermediately susceptible, and three isolates (*P. aeruginosa*, *K. pneumoniae* and *P. pulmonis*) were resistant. FQ resistance is established by point mutations in the DNA gyrase and via plasmid mediated transfer, where genes encoding for quinolone resistance often co-exist on the same plasmid with genes encoding ESBLs [15]. Global surveillance studies illustrate that FQ resistance rates have increased in the past years in almost all bacterial species, where the EU and US now strongly recommend sparing these agents for treatment of more complicated infections [16]. 

Carbapenems remain last-resort antibiotics for treatment of ESBL-producing GNB; however, resistance to these agents is now on the rise. In this study, relatively high susceptibility rates were observed for carbapenem agents in most bacterial species, with few exceptions, where two isolates (*P. aeruginosa* and *C. freundii*) exhibited elevated MICs. Resistance to carbapenems is mainly mediated by the acquisition of carbapenemase enzymes such as OXA or KPC (serine β-lactamase). However, the emergence of Metallo-β-lactamase (MBL) types (e.g., IMP, VIM and NDM-1) in recent years further compounds the problem where they mediate resistance to virtually all β-lactams (except aztreonam) and serine β-lactamase-inhibiting drugs [17], including the novel non-β-lactam β-lactamase inhibitor, avibactam (which is active against AmpC enzymes). MBL screening by the zone enhancement and MTS methods using meropenem plus EDTA, demonstrated that the *P. aeruginosa* isolate was phenotypically positive for MBL. Alarmingly, this isolate also exhibited resistance to the polymyxin class (and every other antibacterial agent tested), where the increasing use of colistin for the treatment of carbapenem-resistant bacterial infections is selecting for resistance. In addition, the unmonitored use of this agent in the agricultural sector is considered a major driver of resistance [18], with the WHO now listing colistin as a crucial last-resort agent. Carbapenem-resistant *P. aeruginosa* is at the top of the WHO priority list, along with carbapenem-resistant *A. baumanii*, and ESBL and carbapenemase-producing Enterobacteriaceae (primarily *E. coli* and *K. pneumoniae*), where there is an urgent and unmet need for development of new antibiotics and infection-control strategies [19]. Certainly, studies conducted highlight the prominence of MDR in these species. Findings further highlight the clinical importance of emerging resistance in non-traditional GNB such as *Citrobacter* spp., Non-*baumannii Acinetobacter* spp., Non-*aeruginosa Pseudomonas* spp. and *Psychrobacter* spp., where high levels of MDR and ESBL production detected in this study correlate with reports in the medical literature [20,21,22,23,24]. The *Proteus* species are additionally emerging as important opportunistic pathogens, where they are now the third most-common etiological factor of UTIs following *Escherichia coli* and *Klebsiella pneumoniae* [25], prompting the WHO to list these species among the Enterobacteriaceae of critical priority. The acquisition of resistance genes in this species is especially worrisome, where species intrinsically harbour resistance to multiple antibiotics including macrolides, tetracyclines, polymyxins and tigecycline. Overall drug-resistant rates of the *Proteus* isolates against other classes of antibacterial agents, such as FQs, extended-spectrum cephalosporins and carbapenems, were low in the present study. However, species were found to harbour the broad-spectrum *bla_TEM-1_* gene, conferring resistance to amoxicillin and narrow spectrum cephalosporins. Additionally, resistance to chloramphenicol was noted for both *P. mirabilis* and *P. penneri*. Another important finding to note in this study was the high level of resistance observed towards azithromycin in the clinical *N. gonorrhoea* isolate, where resistance compromises the current first-line treatment of ceftriaxone (500 mg given intramuscularly as a single dose) combined with azithromycin (2 g given as oral tablet). Azithromycin resistance in *N. gonorrhoea* has seen a stark increase over the last decade [26], when in 2012 guidelines in the EU and elsewhere changed the recommended first-line therapy for gonorrhoea from cefixime (owed to rising resistance) to dual therapy with ceftriaxone and azithromycin [27]. Fortunately, in the present study the *N. gonorrhoea* isolate remained sensitive to all β-lactam agents. However, reports of strains simultaneously resistant to extended-spectrum cephalosporins (including ceftriaxone) and azithromycin have appeared since 2018 [28], where acquisition of resistance to FQs, tetracyclines and aminoglycosides have also been reported. 

To overcome rising resistance, novel alternative therapies to replace or enhance antibiotic agents are required, where phendione offers an alternative option, providing complete inhibition of all test species (Table 2). In recent years, studies have shown the capacity of phendione and its metal complexes as an antimicrobial agent, where findings of this study correlate with those found in the literature. For example, Viganor et al. 2016 described the activity of phendione against both planktonic- and sessile-forming *P. aeruginosa* cells, including cephalosporin- and carbapenem-resistant strains [29]. Authors report MICs that ranged from 3.125 to 12.5 µg/mL, being comparable to those reported for *P. aeruginosa* (15 µg/mL), *P. fulva* (6.25 µg/mL) and *P. fluorescens* (3.125 µg/mL) in the present study. In a more recent study, Ventura et al. 2020 established that significantly lower concentrations of phendione were required to inhibit planktonic carbapenemase-producing *A. baumannii* cells, where reported MICs ranged from 1.56 to 6.25 µg/mL [30]. These results are in accordance with those observed in this study, where *A. beijerinckii*, *A. baumanii* and *A. lwoffii* had respective MICs of 1.56, 0.78, and 0.39 µg/mL. This same research group further investigated the activity of phendione on 46 KPC-producing *K. pneumoniae* isolates [31], where authors report a geometric mean (GM)-MIC of 8.84 µg/mL correlating to an MIC of 6.25 µg/mL for the clinical *K. pneumoniae* isolate in the present study. This same concentration of 6.25 µg/mL was also sufficient for complete inhibition of other Enterobacteriaceae species including *E. coli*, *P. mirabilis* and *P. penneri*, where a lower concentration of 0.39 µg/mL inhibited *C. freundii*, *P. pulmonis* and *N. gonorrhoea*. Phendione is known to exert its antibacterial activity through its avid ability to selectively chelate metal 32ions (e.g., Zn^2+^, Fe^2+^, Ca^2+^, Cu^2+^, Co^2+^, Mn^2+^ and Ni^2+^) necessary for bacterial metabolism and survival [32]. These metal-chelating properties of phendione are proving promising in the treatment of MBL-producing GNB, where MBLs are Zn^2+^-dependent enzymes. However, more in-depth studies investigating the pharmacodynamic and pharmacokinetics properties of phendione are still warranted to complement microbial findings. 

Studies conducted also investigated the capacity of phendione to induce endotoxin release from *E. coli* cells upon treatment, where to the authors knowledge this is the first study to explore this aspect. It is a well-known concept that initiation of antibiotic therapy can aggravate clinical symptoms of infection in patients, where the release of LPS/endotoxin from the outer membrane of GNB can stimulate harmful inflammatory responses in humans. Ceftazidime works by inhibiting penicillin-binding proteins (PBPs) that are essential for cell wall synthesis, where it has a high affinity for PBP-3 (at lower doses) and PBP-1 (at higher doses). Studies have shown that inhibition of PBP-1 causes rapid killing and lysis of bacteria that is associated with low endotoxin release. While binding of ceftazidime to PBP-3 results in the formation of long filaments and subsequent release of large quantities of free endotoxin [33]. Notably, a study conducted by Buijs et al. 2008 showed filament induction can become more extensive (even at higher drug concentrations) during exposure of relatively insusceptible pathogens, including ESBL-producing strains, which can in turn lead to exaggerated release of endotoxin) [34]. Findings of the present study are in agreement with these results, where ceftazidime induced substantial release of endotoxin at all concentrations, where higher drug levels (100 µg/mL) revealed the greatest release of endotoxin per bacterium (up to 384 EU/cfu) (Table 3). In comparison, phendione produced significantly lower endotoxin release per bacterium (8.5 × 10^−4^ EU/cfu) after 3 h treatment at a concentration of 12.5 µg/mL where control cultures produced similar levels (2.7 × 10^−5^ EU/cfu). This suggests that a concentration of 12.5 µg/mL phendione does not significantly induce overall endotoxin release after longer exposure times (of 3 h), though notably it did initially induce higher levels of endotoxin release (7.9 × 10^−3^ EU/cfu at 1.5 h) when compared with the control (4.1 × 10^−5^ EU/mL at 1.5 h). On the other hand, treatment of *E. coli* with 62.5 µg/mL phendione resulted in greater levels of endotoxin release per bacterium (up to 69 EU/cfu), being similar to those at 10 µg/mL ceftazidime (36 EU/cfu). These results provide a valuable insight into phendione-induced endotoxin release, where further studies should additionally investigate other factors such as morphological induced changes due to phendione treatment.

Whilst offering alternative therapeutics is one way of dealing with rising antibiotic resistance, a more useful method to combat the issue is working towards infection prevention. Effective biocidal options play a crucial role in reducing drug-resistant bacteria and their biofilm counterparts that persist on clinical surfaces and the surrounding environment, where the current emergence of biocide-resistant bacterial strains have made it even more difficult to eliminate them [6]. In the present study, all biocidal agents, including peracetic acid, triameen and BAC demonstrated excellent activity against MDR GNB at concentrations recommended by the manufacturer (0.1%) (Table 4 and Figure 2). Notably, peracetic acid proved to be the most efficacious where it was capable of inhibiting *E. coli, A. baumannii*, *P. fulva* and *P. fluorescens* at concentrations of just 0.01% (Figure 2). Studies conducted by this group further show the efficacy of peracetic acid (and triameen) against ATCC strains [35], MDR foodborne Gram-positive and Gram-negative bacteria [36], bacterial spores and various MDR fungal species [37]. Though BAC proved to be an effective agent in this study, many others report rising levels of resistance to this agent [38], owed to its excessive use in health care and community settings. It must also be noted that BAC was the most hindered disinfectant in the presence of interfering substance particularly in the case of the *K. pneumoniae* isolate. Chlorine agents (as well as iodine agents) are known to be impeded by organic matter, where chemical reactions can occur between the BSA protein and the disinfectant, reducing the agent’s overall activity [39]. In addition, BAC was shown to be toxic to the aquatic environment and its inhabitants, where it is increasingly detected in hospital wastewater effluents and other environments [40]. Avoiding injudicious use of biocides that can have a negative impact our agroecosystems is imperative, especially in current times, where the COVID-19 era has led to an enormous surge in their use. This study offers promising alternatives to use of BACs, where peracetic acid is known for its superior environmentally friendly profile. 

## 5. Conclusions

MDR Gram-negative bacteria continue to predominate within hospital wards and communities, where present studies report a high prevalence of MDR and ESBL-production among isolated clinical pathogens. Susceptibility testing highlights the need for improved diagnostics and updated clinical breakpoints, where accurate and timely diagnosis are of upmost importance to patient outcomes. The development of new treatment options is becoming imperative where test agent phendione showed promising potential as an antibacterial agent, providing MICs for all test species. In addition, phendione produced lower levels of endotoxin release at lower concentrations when compared to the β-lactam ceftazidime agent, where further drug biocompatibility testing and pharmacokinetics are necessitated. Effective biocidal agents are indispensable in clinical settings, especially in current times. All test agents in this study provided adequate levels of inhibition at concentrations recommended by the manufacturer, where peracetic acid proved superior, being capable of inhibiting species at lower concentrations.

## Figures and Tables

**Table 1:** Antibiotic profile of test species to a range of antibiotics from varying drug classes with EUCAST target zones given for species for which such information is available. Presence of β-lactamase genes in species is also listed, where ^ denotes broad-spectrum β-Lactamase producer, * denotes ESBL producer, and ** denotes ESBL and AmpC producer.

Drug Class	AG	CHL	Macrolide		Penicillin		Penicillin-Like	Cephalosporins			Monobactam	Carbapenems			Quinolones		Polymyxin		TET	Genes Identified
Antibiotic	S	CHL	AZM	ERY	PEN	AMP	AMC	CTX	CRO	CPD	AZT	DOR	MEM	IPM	CIP	LEV	CT		DOX	
Conc. µg/Disk	10	30	15	15	10	10	20:10	5	30	10	30	10	10	10	5	5	10	50	30	
*A. baumanii* *	13	9	16	9	0 R	8	19	16	17	15	11	29 ^A^ I	25 ^B^ S	30 ^D^ S	30 ^D^ S	32 ^H^ S	0 R	13	10	*blaTEM*
*A. beijerinckii* *	18	21	16	20	0 R	8	19	11	15	13	18	28 ^A^ I	23 ^B^ S	30 ^D^ S	16 ^D^ R	21 ^H^ I	0 R	14	14	*blaTEM*
*A. lwoffii* *	19	25	26	21	13	18	21	17	25	19	8	20 ^A^ R	24 ^B^ S	30 ^D^ S	24 ^D^ S	30 ^H^ S	14	14	25	*blaTEM*
*P. aeruginosa* *	0 R	0 R	0 R	0 R	0 R	0 R	0 R	0 R	0 R	0 R	0 R ^E^	0 R ^A^	0 R ^E^	19 ^H^ R	0 R ^G^	0 R ^A^	14	19	10	*blaTEM*
*P. fulva* *	13	0 R	0 R	0 R	0 R	0 R	8	9	20	0 R	20 ^E^ I	21 ^A^ R	16 ^E^ R	30 ^H^ I	26 ^G^ I	20 ^A^ R	14	18	12	*blaTEM*
*P. fluorescens* *	16	16	14	12	0 R	0 R	15	16	19	13	15 ^E^ R	20 ^A^ R	17 ^E^ R	25 ^H^ I	25 ^G^ R	24 ^A^ S	14	16	19	*-*
*E. coli* **	9	18 ^J^ S	0 R	0 R	0 R	0 R ^L^	12 ^L^ R	16 ^J^ R	17 ^A^ R	15 ^D^ R	12 ^D^ R	21 ^D^ I	23 ^K^ S	20 ^M^ I	24 ^A^ I	24 ^M^ S	13	15	9	*blaTEM, blaAmpC*
*K. pneumoniae* *	8	0 R ^J^	13	0 R	0 R	0 R ^L^	0 R ^L^	22 ^J^ S	21 ^A^ R	16 ^D^ R	19 ^D^ R	25 ^D^ S	26 ^K^ S	30 ^M^ S	10 ^A^ R	13 ^M^ R	12	16	0 R	*blaTEM*
*C. freundii* *	0 R	17 ^J^ R	0 R	0 R	0 R	0 R ^L^	20 ^L^ S	16 ^J^ R	19 ^A^ R	18 ^D^ R	18 ^D^ R	14 ^D^ R	17 ^K^ I	22 ^M^ I	24 ^A^ I	22 ^M^ I	11	15	17	*blaCTX-M1*
*P. pulmonis* *	0 R	13	14	10	0 R	0 R	16	0 R	20	20	0 R	25	27	30	19	19	17	20	15	*blaTEM*
*P. mirabilis ^*	10	18 ^J^	0 R	0 R	10	10 ^L^ R	17 ^L^ S	26 ^J^ S	26 ^A^ S	26 ^D^ S	30 ^D^ S	10 ^D^ R	18 ^K^ I	15 ^M^ R	32 ^A^ S	30 ^M^ S	0 R	0 R	0 R	*blaTEM*
*P. penneri ^*	10	0 R ^J^	0 R	0 R	0 R	0 R ^L^	8 ^L^ R	24 ^J^ S	25 ^A^ S	24 ^D^ S	33 ^D^ S	19 ^D^ R	20 ^K^ I	20 ^M^ I	20 ^A^ R	19 ^M^ I	0 R	0 R	0 R	*blaTEM*
*N. gonorrhoea*	0 R	29	0 R	0 R	27	27	29	27	29	30	29	28	30	40	26	30	0 R	0 R	15	*-*

Abbreviations: AG = aminoglycoside, S = streptomycin, CHL = chloramphenicol, AZM = azithromycin, ERY = erythromycin, PEN = penicillin, AMP—ampicillin, AMC = amoxicillin-clavulanic acid, CTX = cefotaxime, CRO = ceftriaxone, CPD = cefpodoxime, AZT = aztreonam, DOR = doripenem, MEM = meropenem, IPM = imipenem, CIP = ciprofloxacin, LEV = levofloxacin, CT = colistin, TET = tetracycline, and DOX = doxycycline. ^ Broad-spectrum β-Lactamase producer, * ESBL producer, ** ESBL and AmpC producer. EUCAST target zones to indicate susceptibility represented by alphabetical lettering (^A^ = 22 mm, ^B^ = 15 mm, ^D^ = 21 mm, ^E^ = 18 mm, ^G^ = 26 mm, ^H^ = 20 mm, ^J^ = 17 mm, ^K^ = 16 mm, ^L^ = 14 mm, and ^M^ = 19 mm) where S denotes “Susceptible”, I denotes “Susceptible, increased exposure, and R denotes “Resistance” to antibiotic drug.

**Table 2:** MIC results for test species for selected antibiotics and test agent phendione based on EUCAST microdilution plate assay, where MICs for *N. gonorrhoea* were based on gradient test strips and agar dilution method.

Test Species	Antibiotic (µg/mL)														Test Agent (µg/mL)
	ERY	AZM	AMX	AMC	CAZ (3rd)	CRO (3rd)	FEP (4th)	AZT	DOR	MEM	CIP	CHL	DOX	CT	Phendione
*A. baumanii*	32	4	n.d. R	n.d. R	64	64	64	48	0.125 ^A^ S	0.19 ^B^ S	0.19 ^E^ I	128	12	4^A^ R	0.78
*A. beijerinckii*	8	2	32	1	12	32	12	n.d. R	0.094 ^A^ S	0.19 ^B^ S	0.19 ^E^ I	12	6	6 ^A^ R	1.56
*A. lwoffii*	16	1	n.d. R	n.d. R	4	6	1	n.d. R	0.064 ^A^ S	0.125 ^B^ S	0.038 ^E^ S	6	0.5	1.5 ^A^ S	0.39
*P. aeruginosa*	n.d. R	n.d. R	n.d. R	n.d. R	n.d. R ^B^	n.d. R	n.d. R ^B^	n.d. R ^H^	4 ^A^ R	32 ^B^ R	n.d. R ^D^	n.d. R	24	n.d. R^A^	15
*P. fulva*	96	n.d. R	n.d. R	24	n.d. R ^B^	32	24 ^B^ R	n.d. R ^H^	0.5 ^A^ S	1 ^B^ S	0.25 ^D^ I	n.d. R	48	6 ^A^ R	6.25
*P. fluorescens*	64	n.d. R	n.d. R	24	n.d. R ^B^	n.d. R	n.d. R ^B^	n.d. R ^H^	0.19 ^A^ S	0.125 ^B^ S	0.38 ^D^ I	n.d. R	6	12 ^A^ R	3.125
*E. coli*	128	32	16 ^B^ R	16 ^B^ R	1 ^G^ S	3 ^A^ R	1.75 ^G^ S	6 ^G^ R	0.023 ^A^ S	0.023 ^B^ S	0.038 ^D^ S	8 ^B^ S	12	4 ^A^ R	6.25
*K. pneumoniae*	n.d. R	n.d. R	n.d. R ^B^	n.d. R ^B^	6 ^G^ R	2.5 ^A^ R	3 ^G^ S	5 ^G^ R	0.016 ^A^ S	0.5 ^B^ S	n.d. R ^D^	n.d. R ^B^	n.d. R	4 ^A^ R	6.25
*C. freundii*	n.d. R	n.d. R	n.d. R ^B^	12 ^B^ R	4 ^G^ S	n.d. R ^A^	n.d. R ^G^	n.d. R ^G^	32 ^A^ R	4 ^B^ I	0.4 ^D^ I	12 ^B^ R	24	32 ^A^ R	0.39
*P. penneri*	n.d. R	n.d. R	n.d. R ^B^	1.5 ^B^ S	0.064 ^G^ S	0.016 ^A^ S	0.064 ^G^ S	0.047 ^G^ S	0.032 ^A^ S	0.5 ^B^ S	0.075 ^D^ S	n.d. R ^B^	n.d. R	n.d. R ^A^	6.25
*P. mirabilis*	n.d. R	n.d. R	128 ^B^ R	1.5 ^B^ S	0.064 ^G^ S	0.016 ^A^ S	0.064 ^G^ S	0.016 ^G^ S	0.047 ^A^ S	1 ^B^ S	0.023 ^D^ S	24 ^B^ R	n.d. R	n.d. R ^A^	6.25
*P. pulmonis*	n.d. R	n.d. R	n.d. R	1	n.d. R	n.d. R	n.d. R	n.d. R	0.006	0.023	2	n.d. R	64	8	0.39
*N. gonorrhoea*	n.d. R	n.d. R^D^	0.016	0.016	0.16	0.094 ^J^ S	0.047	0.032	0.004	0.023	0.012 ^K^ S	0.5	4	n.d. R	0.39

Abbreviations: n.d. = non determined (concentrations up to 256 µg/mL tested), ERY = erythromycin, AZM = azithromycin, AMX = amoxicillin, AMC = amoxicillin-clavulanic acid, CAZ = ceftazidime, CRO = ceftriaxone, FEP = cefepime, AZT = aztreonam, DOR = doripenem, MEM = meropenem, IPM = imipenem, CIP = ciprofloxacin, CHL = chloramphenicol, DOX = doxycycline, and CT = colistin. EUCAST MIC cutoff points for susceptibility are represented by alphabetical lettering (^A^ = 2 µg/mL, ^B^ = 8 µg/mL, ^D^ = 0.5 µg/mL, ^E^ = 1 µg/mL, ^G^ =4 µg/mL, ^H^ = 16 µg/mL, ^J^ = 0.125 µg/mL, and ^K^ = 0.06 µg/mL) where S denotes “Susceptible”, I denotes “Susceptible, increased exposure, and R denotes “Resistance” to antibiotic drugs.

**Table 3:** Viable counts and endotoxin release for ESBL-producing *E. coli* isolate over a 3 h exposure period to ceftazidime and phendione at the concentrations of 2× MIC, 10× MIC and 100× MIC.

	Treatment	Viable Count (cfu/mL)	Log_10_ Reduction in Viable Count	Endotoxin Release (EU/mL)	Endotoxin/cfu (EU/cfu)
0 h	Control	2.7 × 10^7^		9454	3.5 × 10^−4^
1.5 h	Control	3.6 × 10^8^		22,962	6.4 × 10^−5^
	2× MIC ceftazidime	3.0 × 10^6^	2.1	23,729	7.9 × 10^−3^
	10× MIC ceftazidime	4.0 × 10^5^	3.0	34,797	8.7 × 10^−2^
	100× MIC ceftazidime	2.9 × 10^3^	5.1	34,112	1.2× 10^1^
	2× MIC phendione	3.9 × 10^7^	1.0	39,012	1.0 × 10^−3^
	10× MIC phendione	2.9 × 10^3^	5.1	12,300	4.3 × 10^0^
3 h	Control	9.0 × 10^8^		24,700	2.7 × 10^−5^
	2× MIC ceftazidime	9.0 × 10^5^	3.0	18,824	2.1 × 10^−2^
	10× MIC ceftazidime	1.0 × 10^5^	4.0	35,820	3.6 × 10^1^
	100× MIC ceftazidime	5.5 × 10^1^	7.2	21,122	3.84 × 10^2^
	2× MIC phendione	2.4 × 10^7^	1.6	20,506	8.5 × 10^−4^
	10× MIC phendione	1.5 × 10^2^	6.8	10,300	6.9 × 10^1^

2× MIC ceftazidime = 2 µg/mL; 10× MIC ceftazidime = 10 µg/mL; 100× MIC ceftazidime = 100 µg/mL; 2× MIC phendione = 12.5 µg/mL; 10× MIC phendione = 62.5 µg/mL.

**Table 4:** Zones of inhibition (mm) produced by antimicrobial biocides against Gram-negative MDR isolates in the absence and presence of interfering substance BSA at 3 and 10 g/L (+10 g/L YE) (+/− Standard deviation).

Gram Negative Isolate	BSA (g/L)	Biocide Test Substance								
		BAC			Peracetic Acid			Triameen		
		0.01%	0.1%	1%	0.01%	0.1%	1%	0.01%	0.1%	1%
*E. coli*	0 3 10 *	7.5 (+/−0.1) A 7.4 (+/−0.6) A 7.5 (+/−0.8) A	9.0 (+/−0.7) C 8.5 (+/−0.7) B 9.5 (+/−1.0) C	14.8 (+/−1.0) H 15.0 (+/−1.4) H 14.5 (+/−1.6) H	8.0 (+/−0.1) B 7.8 (+/−0.7) B 7.5 (+/−0.6) A	18.0 (+/−0.9) K 20.4 (+/−1.4) E 20.3 (+/−1.1) E	39.0 (+/−0.7) M 40.5 (+/−0.8) M 39.0 (+/−1.1) M	8.3 (+/−0.4) B 8.0 (+/−0.6) B 7.5 (+/−0.8) A	14.0 (+/−1.2) J 16.5 (+/−0.8) J 16.5 (+/−0.6) J	20.5 (+/−1.4) E 20.4 (+/−0.6) E 20.6 (+/−1.0) E
*K. pneumoniae*	0 3 10 *	0 (+/−0) 0 (+/−0) 0 (+/−0)	7.0 (+/−0.3) A 7.0 (+/−1.0) A 0 (+/−0)	9.8 (+/−0.4) D 9.0 (+/−1.2) C 9.5 (+/−1.2) C	8.0 (+/−0.5) B 8.0 (+/−0.6) B 7.0 (+/−0.6) A	20.3 (+/−1.0) E 20.4 (+/−0.6) E 20.1 (+/−1.1) E	40.0 (+/−0.9) M 34.5 (+/−1.0) N 34.5 (+/−1.2) N	9.0 (+/−0.3) C 9.5 (+/−0.2) C 9.0 (+/−0.7) C	16.0 (+/−1.1) J 16.0 (+/−0.8) J 15.8 (+/−1.0) J	20.3 (+/−0.8) E 20.3 (+/−0.4) E 20.5 (+/−1.0) E
*A. baumannii*	0 3 10 *	8.5 (+/−0.1) B 7.0 (+/−1.0) A 7.0 (+/−1.0) A	14.0 (+/−1.0) 11.0 (+/−0.8) 10.0 (+/0.6) D	20.5 (+/−0.8) E 18.5 (+/−1.0) I 18.9 (+/−1.0) I	9.0 (+/−1.4) C 9.0 (+/−1.1) C 9.5 (+/−0.6) C	20.4 (+/−0.4) 20.3 (+/−1.0) E 20.0 (+/−0.8) E	41.0 (+/−1.0) M 40.5 (+/−0.8) M 40.5 (+/−1.2) M	8.5 (+/−1.2) B 8.3 (+/−1.0) B 8.8 (+/−0.4) C	19.0 (+/−0.9) I 18.9 (+/−0.4) I 19.0 (+/−0.8) I	24.5 (+/−0.8) Q 24.5 (+/−0.6) Q 25.0 (+/−0.2) Q
*P. aeruginosa*	0 3 10 *	0 (+/−0) 0 (+/−0) 0 (+/−0)	10 (+/−0.8) D 7.8 (+/−1.1) B 7.0 (+/−0.7) A	14.5 (+/−1.1) H 10.5 (+/−1.1) D 9.0 (+/−0.8) C	7.5 (+/−0.4) A 7.5 (+/−0.2) A 7.5 (+/−0.3) A	16.5 (+/−1.3) J 16.5 (+/−1.1) J 14.8 (+/−0.4) H	44.3 (+/−1.7) O 38.5 (+/−0.8) M 34.5 (+/−1.5) N	9.5 (+/−0.7) C 8.0 (+/−1.0) B 7.3 (+/−0.4) A	14.5 (+/−0.7) H 14.3 (+/−0.9) H 14.0 (+/−1.8) H	28.0 (+/−0.8) R 19.5 (+/−1.4) E 22.3 (+/−0.8) P
*P. fulva*	0 3 10 *	0 (+/−0) 0 (+/−0) 7.0(+/−0.4) A	10 (+/−0.5) D 9.0 (+/−0.4) C 10 (+/−1.1) D	16.0(+/−0.8) J 16.5(+/−1.1) J 18.0(+/−0.5) I	8.8 (+/−0.3) C 9.5 (+/−0.7) C 9.0 (+/−0.6) C	17.7 (+/−0.4) K 16.8 (+/−1.4) J 17.8 (+/−0.4) K	38.6 (+/−1.4) M 38.4 (+/−1.3) M 34.2 (+/−2.1) N	9.3 (+/−0.4) C 9.5 (+/−0.8) C 10 (+/−0.6) D	16.0 (+/−0.3) J 16.3 (+/−0.5) J 15.8 (+/−1.3) J	22.6 (+/−1.4) P 22.5 (+/−1.1) P 22.5 (+/−0.7) P
*P. fluorescens*	0 3 10 *	7.8 (+/−0.4) B 8.0 (+/−0.1) B 7.0 (+/−0.1) A	11.0 (+/−1.4) F 12.0 (+/−0.1) G 10.0 (+/−0.1) D	19.3 (+/−1.3) I 20.0 (+/−2.1) E 18.5 (+/−1.5) I	10.5 (+/1.8) D 9.0 (+/−1.0) C 9.5 (+/−0.8) C	18.1 (+/−0.7) I 18.0 (+/−1.2) I 16.0 (+/−0.8) J	38.6 (+/−2.1) M 34.3 (+/−1.1) N 34.0 (+/−1.8) N	11.3 (+/−1.4) F 10 (+/−0.4) D 10.8 (+/−1.6) F	22.8 (+/−1.1) P 22.5 (+/−0.5) P 22.5 (+/−0.8) P	30.0 (+/−0.6) T 25.0 (+/−0.9) Q 22.5 (+/−1.2) P
*P. mirabilis*	0 3 10 *	0 (+/−0) 0(+/−0) 0(+/−0)	8.5 (+/−0.7) B 8.0 (+/−0.3) B 8.0 (+/−0.9) B	14.5 (+/−1.5) H 14.8 (+/−1.4) H 14.6 (+/−0.8) H	7.5(+/−0.4) A 8.0(+/−1.0) B 8.5(+/−0.2) B	26.0 (+/−0.9) L 20.0 (+/−1.6) E 20.3 (+/−0.4) E	45.0 (+/−1.7) O 40.5 (+/−1.1) M 40.3 (+/−1.4) M	9.5 (+/−0.4) C 9.0 (+/−0.6) C 10 (+/−1.3) D	26.0 (+/−1.4) L 26.0 (+/−0.2) L 26.0 (+/−0.5) L	33.0 (+/−2.0) S 28.5 (+/−1.8) R 28.5 (+/−0.6) R

* 0 g/L BSA plus 10 g/L yeast extract. Alphabetical lettering indicates there is a significant difference in zone diameter between strains at *p* < 0.05, where groups displaying the same letter are statistically similar whilst those denoted by a different letter are statistically different.

## Data Availability

All data generated or analysed are openly available and included in this published article.

## Supplementary Materials

The following are available online at https://www.mdpi.com/article/10.3390/antibiotics10111283/s1, Table S1: Details of ESBL oligonucleotide primers used in study.
