# Supplementary material for: Effective Antimicrobial Solutions for Eradicating Multi-Resistant and β-Lactamase-Producing Nosocomial Gram-Negative Pathogens
Source: Antibiotics (Basel). 2021 Oct 21;10(11):1283. doi: 10.3390/antibiotics10111283 (PMC8614872; doi:10.3390/antibiotics10111283)
Supplement: Supplementary file 1 [file antibiotics-10-01283-s001.zip › antibiotics-1376458-supplementary.pdf]

Table S1: Details of ESBL oligonucleotide primers used in study

Target Gene	Primer name	Primer Sequence	Expected amplicon size
blaTEM	TEM-F	CATTTCCGTGTCGCCCTTATTC	800bp
	TEM-R	CGTTCATCCATAGTTGCCTGAC	
blaSHV	SHV-F	AGCCGCTTGAGCAAATTAAAC	712bp
	SHV-R	ATCCCGCAGATAAATCACCAC	
blaCTX-M group 1	CTXM1-F	TTAGGAAATGTGCCGCTGTA	688bp
	CTXM1-R	CGATATCGTTGGTGGTACCAT	
blaCTX-M group 2	CTXM2-F	CGTTAACGGCACGATGAC	404bp
	CTXM2-R	CGATATCGTTGGTGGTACCAT	
blaAmpC	AMPC-F	CCCCGCTTATAGAGCAACAA	631bp
	AMPC-R	TCAATGGTCGACTTCACACC	
